# Drying Treatments Change the Composition of Aromatic Compounds from Fresh to Dried Centennial Seedless Grapes

**DOI:** 10.3390/foods10030559

**Published:** 2021-03-08

**Authors:** Hafiz Umer Javed, Dong Wang, Rani Andaleeb, Muhammad Salman Zahid, Ying Shi, Saeed Akhtar, Wang Shiping, Chang-Qing Duan

**Affiliations:** 1 Center for Viticulture & Enology, College of Food Science and Nutritional Engineering, China Agricultural University, No. 17 Tsinghua East Road, Beijing 100083, China; qaziumerjaved@cau.edu.cn (H.U.J.); wangdong@beibingyang.com (D.W.); ying@cau.edu.cn (Y.S.); 2 Key Laboratory of Viticulture and Enology, Ministry of Agriculture, Beijing 100083, China; 3 Department of Plant Sciences, School of Agriculture and Biology, Shanghai Jiao Tong University, 800 Dongchuan Road, Minhang District, Shanghai 200240, China; salmanzahid5@sjtu.edu.cn (M.S.Z.); fruit@sjtu.edu.cn (W.S.); 4 Beijing Yi Qing Food Group Co., Ltd., No. 6 Beixinglu, Dong Duan, Daxing Distruct, Beijing 102600, China; 5 Department of Food Sciences and Engineering, School of Agriculture and Biology, Shanghai Jiao Tong University, 800 Dongchuan Road, Minhang District, Shanghai 200240, China; raniandaleeb2@outlook.com; 6 Institute of Food Science and Nutrition, Bahauddin Zakariya University, Multan 60800, Pakistan; saeedakhtar@bzu.edu.pk

**Keywords:** free-form volatile compounds, glycosidically bound-form volatile compound, centennial seedless, aromatic profile, SPME-GS/MS (solid-phase microextraction condition-gas chromatography/mass spectrometry) 5

## Abstract

Raisin aroma is a vital sensory characteristic that determines consumers’ acceptance. Volatile organic compounds (VOCs) in fresh grapes, air-dried (AD), pre-treated air-dried (PAD), sun-dried (SD), and pre-treated sun-dried (PSD) raisins were analyzed, with 99 and 77 free- and bound-form compounds identified in centennial seedless grapes, respectively. The hexenal, (E)-2-hexenal, 1-hexanol, ethyl alcohol, and ethyl acetate in free-form while benzyl alcohol, β-damascenone, gerenic acid in bound-form were the leading compounds. Overall, the concentration of aldehydes, alcohols, esters, acids, terpenoids, ketones, benzene, and phenols were abundant in fresh grapes but pyrazine and furan were identified in raisin. Out of 99 VOCs, 30 compounds had an odour active value above 1. The intensity of green, floral, and fruity aromas were quite higher in fresh grapes followed by AD-raisins, PAD-raisins, SD-raisins, and PSD-raisins. The intense roasted aroma was found in SD-raisins due to 2,6-diethylpyrazine and 3-ethyl-2,5-dimethylpyrazine. Among raisins, the concentration of unsaturated fatty acid oxidized and Maillard reaction volatiles were higher in SD-raisins and mainly contributed green, fruity and floral, and roasted aromas, respectively.

## 1. Introduction

Raisins are considered to be significant agricultural products due to their high nutritional values. They are a rich reservoir of micronutrients such as vitamins, oligo-elements, polyphenols, and carotenoids. Along with nutritional enhancing qualities, they also boosts immunity against various diseases. These are not only used as nutritional enhancers but also consumed as snacks and are mixed in various foods while cooking, baking, and brewing. China is the third leading raisin producer, with >20 kinds of varieties cultivated for mass production [1]. The central area for raisin production is Turpan, Xinjiang, which accounts for approximately three-quarters of the entire yield of China [2]. Among raisins producing varieties of Turpan, the centennial seedless (white variety) is characterized as a light rose fragrance [3]. Therefore, the centennial seedless variety was selected for studying the aroma compounds due to their unique flavor characteristics.

Raisins are produced by drying grapes. Basically, drying preserves the fruits by the inactivation of the majority of biochemical and physical reactions, the inhibition of microorganism growth, and by slowing down the enzymatic degradation [4]. Solar radiation is a natural source of drying which produces raisins dark in color with a fatty and roasted aroma. In China, raisins are dried by two main methods, i.e., sun-drying and air-drying. Different methods make differences in airspeed, sunlight, and temperature, which ultimately influence the aroma [2,3]. The specific air-drying (AD) technique (hot and dry wind passes through a ventilated and lucifugal house, constructed by claw bricks) is practiced in China, and responsible for generating fruity and floral aromas. On the other hand, the light and temperature intensity are higher in the sun-drying (SD) method that influences the Maillard reaction (MR) and unsaturated fatty acid oxidation (UFAO), producing fatty, roasted, and chemical aromas [5].

Pre-chemical treatments are the most frequently used method that accelerates the drying rate by dissolving the outer wax layer of grapes [6]. Chemical treatment can be carried out using the chemical mixture of alkali, sulfite, acid, and hyperosmotic and gas treatments such as ozone, carbon dioxide, and sulfur dioxide [7]. In order to avoid/minimize the residual chemical additives in the raisin, the chemicals are usually mixed with fat-based oil suspensions such as olive oil or ethyl oleate. Therefore, in this study, the mixture of KOH, Na_2_CO_3_ and lipid is used to quicken the drying process by dissolving the grape skin and increasing the evapotranspiration rate. The concentration, composition, temperature, and pH of the chemicals and time chemical treatments lead to microstructural changes in the fruit skin layers [8]. A series of studies have already been reported in the literature, the impact of pre-chemicals treatments on the rate of drying [9,10], as well as the quality of raisins such as color change, fruit shrinkage [11,12], and volatile compounds in storage [13]. However, no one has studied the comparison of traditional drying methods with or without pre-chemical treatments.

Aroma is a vital sensory characteristic that determines consumers’ acceptance. It arises from many volatile compounds (VOCs) whose composition and concentration vary within different varieties and species [14], vintage, geographical regions, storage conditions, drying methods [2], and type of packing [15]. The Maillard reaction (MR), and the oxidation of lipids (unsaturated fatty acid) and carotenoids are the major sources of raisins and grapes VOCs [16]. The VOCs of fruits are primarily comprised of aldehydes, volatile fatty acids, C13-norisoprenoids, monoterpenes, and alcohols [17]. The hydrolyzation of identified glycosidically bound VOCs in fruits are responsible for the intensified aroma [18]. The aroma produced by free-form VOCs can be sensed directly in grapes or raisins; thus, it is considered a vital component [19]. 

Almost 100 volatile compounds have been qualitatively identified in raisins [2,3,5,13,20,21,22], but many important VOCs have not been identified yet due to lack of delicate isolation and extraction equipment [23]. There has been no study about the evaluation of raisins’ aroma in terms of traditional drying method with or without pre-chemical treatments. Moreover, the volatile components that come from the traditional drying method (air and sun), and pre-chemical treatment might be quite different in concentration and variety. Therefore, there is a need to investigate the free- and glycosidically bound-form aroma compounds of fresh and dried grapes. This information not only leads to the optimization and validation studies to obtain more volatile compounds, but also provides a foundation for further research on the generation mechanism of the VOCs from the Maillard reaction and UFAO. The purpose of this study is to identify and analyze the changes of free and glycosidically bound VOCs during drying and figure out their aroma profile in response to the drying method and pre-chemical treatment.

## 2. Materials and Methods

### 2.1. Experimental Design

A “centennial seedless” grape variety was grown in the territory of the Turpan (Xinjiang province, China), and the fully ripped “centennial seedless” grapes were harvested (total soluble solids, TSS >20 °Brix) in October 2014. The bunches of 600 kg grapes were divided into two parts, and then one cluster of grapes was soaked in a drying promoter (lipid, KOH, and Na_2_CO_3_) for one minute. Thereafter, the unique air-drying and traditional sun-drying methods were practiced, making the raisins from the fully matured grapes (treated and/or non-treated). The drying course continued until the moisture content (<15%) remained constant for three days. The desired moisture content obtained regarding treatments such as AD (air-dried), PAD (pre-treated air-dried), SD (sun-dried), and PSD (pre-treated sun-dried) took 43, 21, 19, and 12 days, respectively (Figure S1). The 1 kg samples were collected from fresh grapes ˃85% and dried grapes (raisin) ˂15%, then packed in plastic bags in triplicate and transported to the lab with dry ice covering in air freight. 

### 2.2. Chemicals

The description of all chemicals used in this study is mentioned in File S1.

### 2.3. Sample Pre-Treatment

The preparation of samples was carried by minor modifications in the procedure of Wang et al. [2]. The raisins (100 g) were dipped in an equal amount of double distilled water and kept for 12 h at 4 °C. After that, they were macerated and homogenized for 240 min. Then, the pulp sample was centrifuged thrice (4 °C and 8000 rpm for 10 min) until the complete superimposition was attained [24]. The clear floating liquid was utilized for the detection of free- and bound-form compounds.

### 2.4. Preparation of Free- and Glycosidically Bound-Form Volatiles 

The clear floating fluid was directly used for the detection of free-form compounds. In contrast, the method of Wen et al. [25] was used for the detection of glycosidically bound volatilities with minor amendments. All samples were run in triplicate. 

The Cleanert (PED-SPE) column was employed to detach the glycosidically bound VOCs. Firstly, the column was activated with water (10 mL) and methanol (10 mL) separately, and then, 1 mL of the sample was added. The majority of the free-form VOCs were removed by 5 mL of dichloromethane, whereas the sugar and acids were rinsed by water (5 mL). After that, glycosidically bound compounds were eluted in a 50 mL flask using 20 mL of methanol. Finally, the bound-form VOCs were obtained by removing the solvent with a rotary evaporator (30 °C). Subsequently, the bound-form compounds were enzymatically hydrolyzed by pouring 5 mL of citrate/phosphate buffer solution (2 M; pH 5) into a vial. The process was carried out for 16 h (40 °C) in an incubator with the action of AR2000 [25,26].

### 2.5. Solid-Phase Microextraction Condition (SPME)

The both (free- and bound-form) VOCs of raisins were extracted by following solid-phase microextraction condition (SPME) situations: 5 mL of clear supernatant and 10 μL of internal standard (4-methyl-2-pentanol; 1.0018 mg/L) were mixed in a flask holding a magnetic stirrer. Then, NaCl (1.3 g) was poured, and the flask was tightly plugged with a silicon base polytetrafluoroethylene (PTFE) stopper and then equilibrated on a hotplate (60 °C; 40 min) with constant agitation. After that, the VOCs were extracted by extraction coating fiber Carboxen/Polydimethylsiloxane/ Divinylbenzene (CAR/PDMS/DVB) in the headspace of the flask for 40 min. Finally, the fiber was desorbed into the gas chromatography (GC) injection port for 8 min.

### 2.6. GC/MS Analysis

The isolation and detection of the aromatic compounds of the raisins were executed in a GC-7890 attached with a MS-5975 and fitted out with a 60 m × 0.25 mm (HP-INNOWax) capillary column with a film thickness of 0.25 μm (J&W Scientific, Folsom, CA, USA). The condition of the GC/MS temperature was similar to our previous study [2,5]. The carrier gas “helium” was adjusted at a flow rate (1 mL per min) with the splitless GC inlet mode for SPME extract injection. The oven temperature was adjusted, as mentioned in our previous studies [2,5,13].

Mass spectra were achieved in an electron impact manner with an ionization energy and source temperature of 70 eV and 230 °C, respectively. The mass range (20–450 *m*/*z*) was used as full-scan mode, and then the process proceeded with an auto-tuning condition for the selective ion mode. The retention indices (RI) were obtained by the retention time (RT) of C6–C24 n-alkane in similar chromatographic circumstances. Identification was based on the RI of available standards and matching with mass spectra in the NIST-08 library. Wherein the unavailability of reference standards, a tentative determination was performed by comparing mass-spectra with NIST-08 library and retention indices present in the aforementioned literature.

### 2.7. Quantification of VOCs

The method of quantification was followed as reported by Wu et al. [27], with little modification. The standard amount of the acids and sugars in the raisin supernatant was used to prepare an artificial raisin suspension. The simulated solution was prepared by adding 5 g/L of tartaric acid and 400 g/L of sugars (glucose) in distilled water, and then a 4.2 pH was maintained with 1 M of sodium hydroxide solution. For the spike, the standard compounds (known concentration) were mixed in HPLC-grade ethanol and were further diluted into two-fold dilution with 15 levels by using an artificial raisin suspension. The 15 levels of standard VOC solutions were run in a similar way to the raisin supernatant. The regression coefficients of calibration curves quantified the aroma compounds at above 0.99. Without standard compounds, the contents of volatiles were measured by comparison with the standard curves holding a similar number of C-atoms or identical functional group. The quantitative data of the identified VOCs were checked by particular ion peak areas regarding the internal standard. 

### 2.8. OAVs Calculation

Odor activity value (OAV) is the proportion of the VOCs content to its odor threshold in a water-based solution, and compounds with >1 OAVs are considered as flavor contributors (aroma compounds) [16,28]. The identified aroma compounds were classified into six standard classes based on flavor such as floral, green, fruity, fatty, roasted, and chemical aromas. The OAVs were calculated by following previously reported criteria [2,19,29].

### 2.9. Statistical Procedures 

The data analysis was executed using SPSS software. A one-way ANOVA test was used to analyze the significant impact of different drying treatments on VOCs, employing least significant difference LSD tests at *p* < 0.05. The principal component analysis was executed under the concentration of UFAO and MR volatiles using R-software.

## 3. Results and Discussion

A total of 100 VOCs were successfully identified, including 14 aldehydes, 19 alcohols, 16 esters, nine acids, 22 terpenes, seven ketones, five furans and benzenes, four pyrazines, and two phenols, of which three VOCs (α-Terpinene, geranial, and 2,3-diethyl, pyrazine) had not been previously reported in raisins (Table S1). The remaining 97 compounds were mentioned in different studies regarding raisin varieties, drying treatments, and storage [2,3,5,13,20,21,22]. The concentration (average ± standard deviation) of both (free- and bound-form) VOCs of the different treats (AD, PAD, SD, and PSD) are presented in Table S1. There were 99 and 77 compounds that existed in free- and bound-form, respectively. The additional 22 VOCs subsisted only in the free-form because they can not be linked to the sugar through the ‘‘glycosidic bond’’ deprived of similar groups [2].

**Figure 1:**
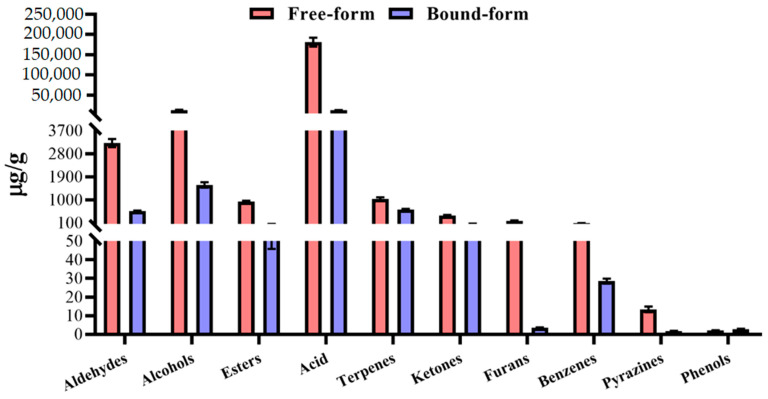
The concentration of free- and glycosidically bound-form compounds in different classes of centennial seedless.

Overall, the concentration of bound-form VOCs in aldehydes, alcohols, esters, acids, terpenes, ketones, furans, benzenes, and pyrazines was significantly lower than free-form volatiles (Figure 1). The hydrolyzation of glycosidically bound compounds is the reason for less volatile content, which changes into an intense flavor [18]. Only the phenolic group had more content in bound-form as a result of the higher content of 4-vinylguaiacol. Some compounds such as 3-methyl-2-buten-1-ol, ethyl decanoate, and 2,3-diethyl, pyrazine only existed in bound-form, which might be due to the strong glycosidic bounding of the sugar with hydroxide group. Among the bound-form volatiles, pentanal, hexanal, 3-methyl-1-butanal, nonanoic acid, acetic acid, and geranic acid had a concentration above 500 µg/g (Table S1). The concentration of all bound-form compounds was more plentiful in fresh grapes than dried, except styrene, 1-hexanol, and nonanal. The compounds such as octanal, methyl hexadecanoate, and 2-acetylfuran were only found in fresh grapes. At the same time, 1-butanol, ethyl octanoate, ethyl decanoate, (E)-2-nonenal, and (E,E)-2,4-heptadienal were not identified in fresh grapes (Table S1). 

### 3.1. Free-Form Compounds

#### 3.1.1. Aldehydes

The concentration of all aldehyde compounds was higher in fresh grapes, except (E,E)-2,4-nonadienal, (E)-2-heptenal, and benzaldehyde. The compounds (E)-2-nonenal and (E,E)-2,4-heptadienal came from the oxidation of linoleic acid [30], and decanal produced by the oxidation of oleic acid [31] were not identified in fresh grapes (Table 1). The content of VOCs was comparatively higher in fresh grapes as compared to all raisins, which were prepared in different ways. Among raisins, the SD and PSD treatments were produced in higher concentrations than AD and PAD, excluding (E)-2-heptanal and phenylacetaldehyde (Table 1). The higher content in SD and PSD is might be due to the intense light with high temperature [3,5].

#### 3.1.2. Alcohols

The most concentrated alcoholic compounds, such as (Z)-3-hexen-1-ol and 1-hexanol which provide the green aroma while 1-butanol generate the fruity smell, were significantly much higher in fresh grapes than the dried raisins (AD, PAD, SD, and PSD). The 2-octanol and 2-nonanol only existed in fresh grapes, whereas, 3-methyl-1-butanol were generated in all forms of dried raisins (Table 1). The concentration of 1-pentanol, 1-hexanol, 1-heptanol, 1-octanol, 2-ethyl-1-hexanol, (Z)-3-hexen-1-ol, sulcatol, and 1-butanol in fresh grapes, 2-octen-1-ol and 3-methyl-1-butanol in AD-raisins, and 1-octen-3-ol in PSD-raisins were relatively higher.

#### 3.1.3. Ester

The ethyl hexanoate, γ-nonalactone, and ethyl acetate provided the fruity note to the raisins. Their concentration was significantly higher in PAD-raisins than fresh grapes, AD-raisins, SD-raisins, and PSD-raisins. The volatiles, such as ethyl nonanoate and methyl salicylate, were only identified in PAD-raisins (Table 1). Among raisins, methyl hexadecanoate and β-Ionone existed in air-dried raisins while diethyl succinate was specified to pre-chemical treated raisins. 

#### 3.1.4. Acids

The pentanoic acid, hexanoic acid, and octanoic acids were produced by the oxidation of methyl linoleic acid [32] and with a higher concentration in SD-raisins, fresh grapes, and AD-raisins, respectively. Dodecanoic acid and 2-ethyl hexanoic acid were only identified in PAD-raisins with a concentration of more than ten µg/g (Table 1). 

#### 3.1.5. Terpenoids

Terpenoid is one of the leading classes in this study, markedly providing fruity and floral flavor to fruits [33]. It is also responsible for the fragrance in both grapes and raisins [2]. All terpenes compounds were significantly higher in fresh grapes as compared to dried raisins, except nerol oxide, and nerolidol 2, lilac alcohol, and cosmene didn’t quantify in grapes. Among raisins, the amount of VOCs was comparatively higher in AD-raisins, and least were recorded in SD-raisins (Table 1). The high temperature and light intensity in the sun-drying method might cause less concentration in terpenes [3,5]. Geraniol, geranial, linalool, and β-damascenone were significantly assisted in the fruity and floral aroma of raisins [34].

#### 3.1.6. Furans and Pyrazines

Except 2-pentyl furan, all other furan compounds come from the Maillard reaction [35] during the drying and storage of raisins [3,5], and they were not found in the fresh grapes. The 2-pentyl furan, furfural, and 2-acetylfuran in SD-raisins, and the 5-methyl-2-furfural and 5-hydroxymethyl-2-furaldehyde in PSD-raisins were significantly greater than AD- and PAD-raisins (Table 1).

There were three pyrazine VOCs, 2-ethyl-6-methyl pyrazine, 5-ethyl-2,3-dimethyl pyrazine, and 2,6-diethylpyrazine which were already reported in raisins [2,21]. During the drying process, the pyrazine compounds are produced through the Maillard reaction and are responsible for a strong roasted aroma to dried grapes. 2-Ethyl-6-methylpyrazine was significantly higher in fresh grapes and could be originated by the condensation process of aspartic acid with galactose, fructose, and glucose [35]. Moreover, 2,6-diethyl pyrazine and 5-ethyl-2,3-dimethylpyrazine were generated from the reaction of aspartic acid and [36], and their amount was significantly higher in SD-raisins but was not found in fresh grapes (Table 1). 

#### 3.1.7. Ketones, Benzenes and Phenol 

All the ketone compounds found in this study were also previously observed in dried grapes [2,21,22]. They did not majorly contribute to aroma [2] due to the high threshold odor value. 3-Octen-2-one, 2,3-butanedione, and acetoin did not exist in fresh grapes, and their concentration was significantly higher in SD-raisins following PSD-raisins, AD-raisins, and PAD-raisins (Table 1).

A high amount of benzene compounds were noted in fresh grapes. Among raisins, styrene, o-cymene, and toluene were statistically at par while naphthalene and 2-methylnaphthalene had higher amounts in AD-raisins. The identified benzene compounds in grapes and raisins produced a chemical note and it cannot contribute to aroma due to the low concentration and high threshold, as mentioned in the previous study [3]. Only two phenolic compounds are identified in fresh and dried grapes, whichhave already been reported in three raisin varieties dried by air [2]. The 4-ethenyl-2-methoxyphenol was produced from the breakdown of ferulic acid [37] and was only identified in fresh centennial grapes, but it cannot be found in raisins as mentioned in earlier studies [38].

### 3.2. Effect of Drying Method on UFAO- and MR-VOCs 

**Figure 2:**
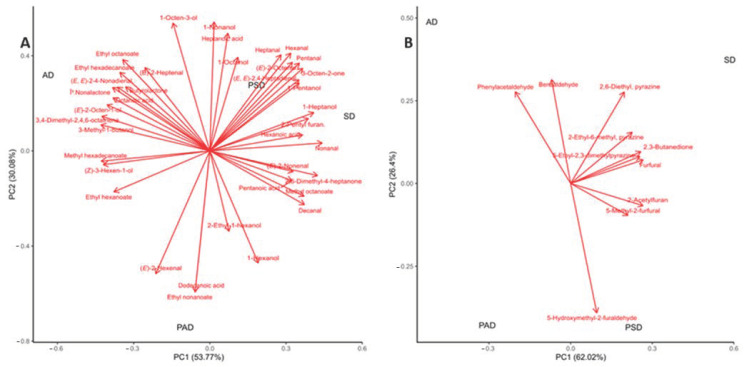
Principal components analysis (PCA) plot based on UFAO and MR volatile compounds in dried raisins. SD: Sun-drying; PSD: pre−treated sun−drying; AD: Air−drying; PAD: pre−treated air−drying. (**A**) Unsaturated fatty acid oxidation (compounds); (**B**) Maillard Reaction (MR) compounds.

The 41 and 10 VOCs of UFAO and MR, respectively, were analyzed using principal components (PCs) analysis to determine the effect of different drying treatments (AD, PAD, SD, and PSD). Regarding drying treatments, the first two PCs of UFAO and MR corresponded to 83.85% and 88.42%, respectively. The UFAO compounds were well separated regarding drying treatments. The majority of the UFAO compounds belonged to SD and PSD raisins following AD and PAD raisins (Figure 2A). Of the total variance of PC1, 62.02% was characterized by MR compounds, and they were closely linked to SD treatments (Figure 2B). The phenylacetaldehydes and benzaldehyde were considered to belong to AD, while 5-hydroxymethyl-2-furaldehyde belonged to PSD methods.

### 3.3. Aroma Profile

Out of 99 free-form VOCs, 30 were sensed as flavor compounds in fresh grapes and dried raisins. Their OAV (odour active value), TVW (threshold value in water) aroma series, and aroma descriptor are mentioned in (Table 2). The 23, 24, 29, 24, and 26 aromatic compounds (OAVs ≥ 1) were identified in fresh grapes, AD-raisins, PAD-raisins, SD-raisins, and PSD-raisins, respectively. The concentration level of geranic acid, (Z)-3-hexen-1-ol, (E,E)-2,4-heptadienal, geranial, geraniol, α-terpineol, 3-methyl-1-butanol, and acetic acid were consequently so high that they surpassed the threshold greatly. The content of β-damascenone, β-ionone, (E)-2-nonenal, and rose oxide were not so high; however, their TVW is low enough to give a compelling aroma.

**Figure 3:**
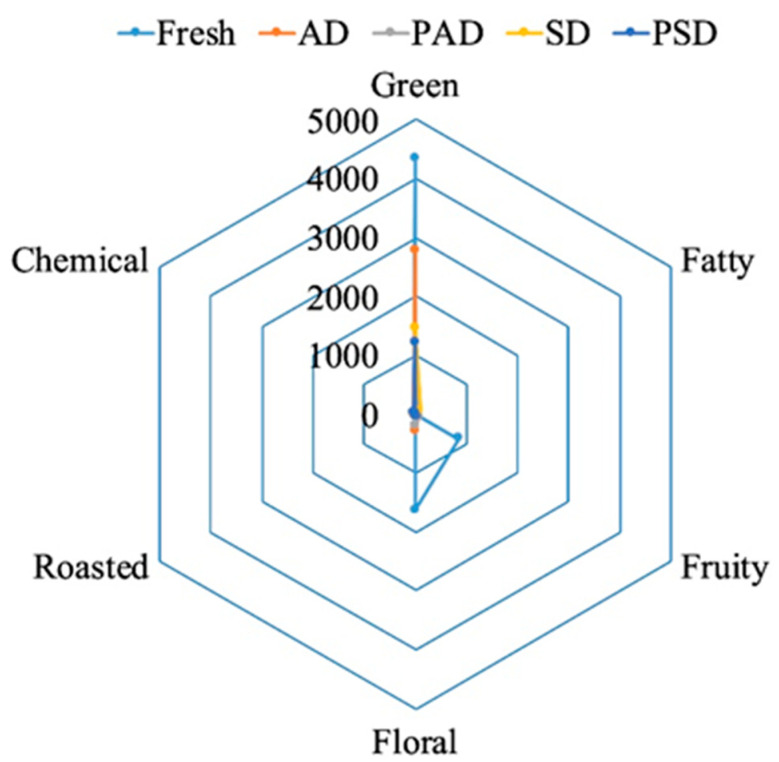
The aromatic series of free-form VOCs in fresh and dried grapes based on odor activity values (OAVs). AD (air-dried); PAD (pre-treated air-dried); SD (sun-dried); PSD (pre-treated sun-dried).

The aromatic series of centennial seedless as fresh and dried (AD, PAD, SD, and PSD) grapes are presented in Figure 3. Based on aroma character, the 30 aroma-producing compounds were classified into six categories, including green, fatty, roasted, chemical, fruity, and floral aromas.

The flavor characteristics (green, fruity, floral, and chemical) were relatively higher in fresh grapes than dried grapes. Among raisins, the strength of fruity and floral fragrances was abundant in AD-raisins as compared to others. Correspondingly, floral and fruity (tropical tree fruit) is the main character of AD-raisins in storage [5], as well as in sweet wine prepared by Garnacha Tintorera grapes [40]. The β-damascenone (threshold value 0.09 µg/L) was the major contributor of floral and fruity notes [2,41]. Besides, β-ionone is responsible for the floral aroma and their intensity was higher in fresh grapes, AD-raisins and PAD-raisins, was not generated in SD- and PSD-raisins. The terpene compounds, especially geraniol and linalool, β-damascenone, and β-ionone, were reported as a major contributor to the fruity and floral aroma in litchis [27], blackberries [42], and raisins [2,3]. The green aroma had the most intense aroma as compared to fruity, floral, roasted, and chemical and they were mainly strengthened by hexenal, (E,E)-2,4-heptadienal and octanal, particularly higher in fresh grapes. Whereas, the roasted aroma came from MR during the drying of the grapes, mainly produced through 2,6-diethylpyrazine and 3-ethyl-2,5-dimethylpyrazine, and were more intense in SD-raisins but did not contribute to fresh grapes. These two volatile compounds were found as outstanding aromatic compounds in roasted coffee [43] and dried raisins [2].

## 4. Conclusions

Volatile compounds in fresh grapes and dried raisins (AD-raisins, PSD-raisins, SD-raisins, and PSD-raisins) were detected and evaluated. The terpenoids and aldehydes were the main class of VOCs that were responsible for the aroma in both grapes and raisins. The aroma profile in fresh grapes and dried raisins was quite different. The main characteristic aroma was green, fruity, and floral, which were mainly produced in fresh grapes and AD-raisins, while the intensity of roasted and fatty aromas was more compelling in SD-raisins. Further studies will focus on the model reaction of UFAO and MR during the drying of grapes.

## Figures and Tables

**Table 1:** The concentration (µg/L)A of free-form volatile compounds in the fresh and dried centennial seedless grapes.

S.N	RI	Compound Name	Ion *m*/*z*	ID	Source	Fresh	AD-Raisin	PAD-Raisin	SD-Raisin	PSD-Raisin
Aldehydes										
1	975	Pentanal ^W,J^	44	2	UFAO	167.92 ± 13.06a	69.36 ± 2.34cd	64.23 ± 2.51d	86.8 ± 6.14bc	81.01 ± 7.64b
2	1066	Hexanal ^B,W,J^	44	1	UFAO	6972.72 ± 365.7a	365.44 ± 24.86b	285.95 ± 2.96b	547.62 ± 13.12b	551.07 ± 5.42b
3	1178	Heptanal ^B,W,J^	44	2	UFAO	86.1 ± 7.32a	34.22 ± 0.87bc	24.76 ± 1.43c	48.17 ± 3.71b	36.66 ± 0.74bc
4	1292	Octanal ^B,W^	43	2	UFAO	386.85 ± 45.07a	NF	NF	NF	NF
5	1393	Nonanal ^B,W,J^	57	1	UFAO	6.38 ± 0.08a	1.18 ± 0.23c	1.38 ± 0.07bc	1.74 ± 0.35b	1.49 ± 0.27bc
6	1501	Decanal ^B,W,J^	43	1	UFAO	NF	NF	1.88 ± 0.03a	1.84 ± 0.07a	2.08 ± 0.44a
7	1217	*(E)*-2-Hexenal ^B,W,J^	41	1	UFAO	3304.21 ± 85.45a	63.7 ± 3.77c	134.68 ± 9.12b	17.77 ± 0.32c	44.97 ± 2.29c
8	1325	*(E)*-2-Heptenal ^B,W,J^	41	2	UFAO	27.41 ± 2.29c	111.29 ± 6.05a	18.41 ± 0.37c	57.83 ± 9.67b	24.88 ± 3.41c
9	1434	*(E)*-2-Octenal ^B,W,J^	41	2	UFAO	22.7 ± 0.75a	6.69 ± 1.41d	4.68 ± 0.16e	11.39 ± 1.92b	8.38 ± 0.92c
10	1539	*(E)*-2-Nonenal ^B,W^	43	2	UFAO	NF	NF	2.5 ± 0.1b	7.44 ± 0.38a	NF
11	1497	*(E,E)*-2,4-Heptadienal ^B,W,J^	81	2	UFAO	NF	NF	NF	86.71 ± 4.17b	111.18 ± 7.03a
12	1705	*(E,E)*-2,4-Nonadienal ^B,W,J^	81	2	UFAO	179.65 ± 3.2b	241.35 ± 13.59a	95.46 ± 8c	106.78 ± 12.96c	94.33 ± 4.12c
13	1530	Benzaldehyde ^B,W,J^	77	1	MR	13.43 ± 0.13b	25.6 ± 1.57a	15.2 ± 0.64b	14 ± 1.75b	14.05 ± 1.46b
14	1650	Phenylacetaldehyde ^B,W,J^	91	1	MR	386.96 ± 11.48a	326.67 ± 21.3b	163.4 ± 23.81c	141.21 ± 11.87d	101.89 ± 9.17e
Alcohols										
15	1205	1-Pentanol ^W,J^	52	2	UFAO	14.81 ± 0.74a	8.54 ± 0.77c	8.69 ± 0.28c	11.92 ± 0.66b	12.99 ± 0.43b
16	1349	1-Hexanol ^W,J^	56	2	UFAO	901.25 ± 76.68a	74.36 ± 4.56c	156.45 ± 7.01b	110.57 ± 11.26bc	128.71 ± 2.79bc
17	1453	1-Heptanol ^W,J^	70	2	UFAO	9.65 ± 0.22a	NF	2.21 ± 0.01c	7.13 ± 0.95b	7.58 ± 0.83b
18	1555	1-Octanol ^B,W,J^	56	1	UFAO	6.71 ± 0.22a	2.64 ± 0.19c	1.37 ± 0.05d	2.63 ± 0.46c	5.55 ± 0.26b
19	1657	1-Nonanol ^W,J^	56	1	UFAO	1.93 ± 0.06a	0.58 ± 0.01bc	0.32 ± 0.01d	0.51 ± 0.06c	0.7 ± 0.12b
20	1411	2-Octanol ^B,W,J^	45	2	UFAO	0.83 ± 0.06a	NF	NF	NF	NF
21	1488	2-Nonanol ^J^	45	2	UFAO	1.86 ± 0.07a	NF	NF	NF	NF
22	1449	1-Octen-3-ol ^B,W,J^	57	1	UFAO	14.93 ± 0.62d	23.18 ± 3.3c	24.99 ± 0.8c	36.48 ± 5.83b	52.42 ± 2.3a
23	1614	(E)-2-Octen-1-ol ^W,J^	57	2	UFAO	3.15 ± 0.07d	10.86 ± 0.09a	5.65 ± 0.34c	2.5 ± 0.57e	7.42 ± 0.34b
24	1487	2-Ethyl-1-hexanol ^W,J^	57	1	UFAO	15.26 ± 0.41a	0.78 ± 0.08c	3.31 ± 0.01b	1.03 ± 0.51c	3.24 ± 0.15b
25	1395	(Z)-3-Hexen-1-ol ^J^	67	2	UFAO	387.23 ± 28.45a	52.3 ± 0.71b	38.83 ± 0.74b	NF	32.11 ± 0.19b
26	1203	3-Methyl-1-butanol ^W,J^	55	1	UFAO	NF	1814.98 ± 32.3a	1455.15 ± 26.93b	1130.05 ± 32.01c	1487.78 ± 24.13b
27	1458	Sulcatol ^J^	95	2	CR (carotenoid)	9.84 ± 0.14a	2.73 ± 0.04b	1.68 ± 0.03c	0.74 ± 0.23d	1.44 ± 0.16c
28	942	Ethyl alcohol ^J^	31	2		13,143.64 ± 1679ab	13,526.01 ± 531a	7751.14 ± 85.13c	8197.47 ± 165c	11,920.23 ± 732d
29	1317	3-Methyl-2-buten-1-ol ^W,J^	71	2		NF	NF	NF	NF	NF
30	1879	Benzyl alcohol ^W,J^	79	1		279.02 ± 18.52a	283.38 ± 16.54a	109.02 ± 3.61b	23.96 ± 0.05c	120.75 ± 5.05b
31	1914	Phenylethyl alcohol ^W,J^	91	2		363.21 ± 32.76a	358.05 ± 37.8a	92.78 ± 2.82c	43.41 ± 6.67d	155.29 ± 13.89b
32	1148	1-Butanol ^J^	56	2		248.09 ± 35.63a	47.43 ± 1.51b	44.66 ± 0.93b	37.55 ± 4.11b	32.58 ± 2.4b
Esters										
34	1227	Ethyl hexanoate ^W,J^	88	1	UFAO	10.63 ± 2.55b	14.38 ± 4.31a	11.67 ± 0.21b	6.32 ± 2.87	3.82 ± 1.48d
35	1570	Ethyl octanoate ^W,J^	88	1	UFAO	5.56 ± 0.45a	2.43 ± 0.21b	1.13 ± 0.03d	1.17 ± 0.07d	1.72 ± 0.13c
36	1378	Methyl octanoate ^J^	74	2	UFAO	NF	NF	0.14 ± 0b	0.14 ± 0.01b	0.17 ± 0.02a
37	1570	Ethyl nonanoate ^J^	88	1	UFAO	NF	NF	0.16 ± 0a	NF	NF
38	1227	Ethyl hexadecanoate ^W,J^	88	1	UFAO	47.55 ± 5.31a	11.39 ± 0.54b	7.71 ± 0.28b	8.13 ± 0.09b	8.29 ± 0.09b
39	2163	Methyl hexadecanoate ^J^	74	2	UFAO	0.44 ± 0.06a	0.14 ± 0.01b	0.07 ± 0c	NF	NF
40	1636	Butyrolactone ^W,J^	42	2	UFAO	3.75 ± 0.5d	8.68 ± 0.12a	6.58 ± 0.52b	5.57 ± 0.4c	8.19 ± 0.74a
41	2035	γ-Nonalactone ^W,J^	85	2	UFAO	3.34 ± 0.29b	8.99 ± 0.68a	1.49 ± 0.04c	1.43 ± 0.12c	1.81 ± 0.57c
42	885	Ethyl acetate ^W,J^	43	1		322.94 ± 20.33d	1509.48 ± 34.63a	1400.73 ± 63.66b	368.82 ± 16.62d	801.43 ± 22.32c
43	1677	Diethyl succinate ^W,J^	101	1		NF	NF	1.57 ± 0.04a	NF	1.46 ± 0.06b
44	1782	Methyl salicylate ^J^	120	1		NF	NF	2.98 ± 0.09a	NF	NF
45	1253	Ethyl salicylate ^J^		2		NF	NF	0.54 ± 0.01a	0.36 ± 0.01b	NF
46	942	Ethyl alcohol ^J^	31	2		13,143.64 ± 1679.4ab	13,526.01 ± 531.25a	7751.14 ± 85.13c	8197.47 ± 165.51c	11,920.23 ± 732.92b
47	1470	β-Ionone ^J^	177	2	CR	5.41 ± 0.21a	1.07 ± 0.05b	0.75 ± 0.02c	NF	NF
Acids										
48	1740	Pentanoic acid ^W,J^	60	2	UFAO	NF	NF	74.41 ± 2.76b	187.82 ± 8.88a	NF
49	1847	Hexanoic acid ^B,W,J^	60	1	UFAO	375.48 ± 19.8a	153.07 ± 13.89b	161.91 ± 26.48b	214.5 ± 11.34b	161.37 ± 56.99b
50	1953	Heptanoic acid ^B,W,J^	60	1	UFAO	3.19 ± 0.57a	2.58 ± 0.17ab	2.17 ± 0.11b	2.52 ± 0.35ab	2.96 ± 0.66a
51	2060	Octanoic acid ^B,W,J^	60	1	UFAO	2.41 ± 0.16b	4.63 ± 0.19a	2.33 ± 0.22b	2.29 ± 0.12b	2.11 ± 0.72b
52	2166	Nonanoic acid ^B,W,J^	60	2		20.16 ± 1.9a	4.45 ± 0.17c	8.69 ± 1.26b	3.53 ± 0.03c	4.76 ± 0.24c
53	2484	Dodecanoic acid ^W,J^	73	2	UFAO	NF	NF	10.9 ± 2.72a	NF	NF
54	1950	2-Ethylhexanoic acid ^W,J^	88	1		NF	NF	28.97 ± 0.51a	NF	NF
55	1433	Acetic acid ^J^		2		135,518.4 ± 11152.8d	265,800.8 ± 12361.5a	180,758.57 ± 7545.9c	209,013.96 ± 17456.5b	114,244.24 ± 6693.1e
Terpenes										
56	1011	α-Terpinene ^N^	121	2		377.96 ± 11.76a	36.38 ± 3.67b	18.52 ± 0.64c	NF	19.33 ± 2.3c
57	1286	Terpinolene ^J^	93	1		185.51 ± 8.78a	17.59 ± 0.51b	14.86 ± 0.16bc	9.47 ± 0.09c	NF
58	1473	Nerol oxide ^W,J^	68	2		141.28 ± 6.83b	226.12 ± 6.18a	65.67 ± 0.56d	22.77 ± 4.76e	99.44 ± 6.57c
59	1548	Linalool ^W,J^	71	1		247.23 ± 32.98a	15.44 ± 0.71b	13.63 ± 0.49b	4.81 ± 0.39b	4.9 ± 0.69b
60	1610	Hotrienol ^W,J^	71	1		99.66 ± 2.9a	31.05 ± 0.44b	25 ± 0.43c	14.51 ± 0.82d	10.67 ± 0.16e
61	1620	p-Menth-1-en-9-al ^W,J^	94	2		40.38 ± 0.5a	11.18 ± 0.09b	6.55 ± 0.12c	6.36 ± 0.32cd	5.77 ± 0.43c
62	1698	α-terpineol ^B,W,J^	59	1		77.53 ± 2.74a	12.66 ± 0.6b	9.63 ± 0.13c	6.36 ± 0.15d	7.29 ± 0.45d
63	1739	cis-Pyran linalool oxide ^W,J^		2		8.69 ± 0.31a	6.28 ± 0.08b	2.86 ± 0.08c	2.69 ± 0.07c	2.45 ± 0.23c
64	1764	β-Citronellol ^J^	69	1		7.62 ± 0.28a	2.84 ± 0.15b	1.79 ± 0.45c	NF	NF
65	1799	Nerol ^W,J^	69	2		276.43 ± 3.01a	74.14 ± 3.33b	26.37 ± 0.15d	3.22 ± 0.18e	30.93 ± 2.77c
66	1825	β-damascenone ^W,J^	69	1		62.93 ± 7.23a	3.26 ± 0.13b	3.21 ± 0.1b	2.21 ± 0.07b	2.26 ± 0.4b
67	1847	Geraniol ^W,J^	69	1		235.23 ± 2.38a	71.45 ± 6.14b	21.77 ± 0.09c	1.54 ± 0.15d	25.63 ± 2.38c
68	2049	Nerolidol 2 ^J^	41	2		NF	NF	0.58 ± 0a	NF	NF
69	2340	Geranic acid ^W,J^	69	1		489.07 ± 56.92a	149.32 ± 5.42b	86.18 ± 6.87c	16.74 ± 0.4d	25.57 ± 1.4d
70	1857	Geranylacetone ^B,W,J^	43	1	CR	6.1 ± 0.74a	1.4 ± 0.52b	0.8 ± 0.07b	0.77 ± 0.07b	1.18 ± 0.1b
71	1132	3,4-Dimethyl-2,4,6-octatriene ^J^	121	2	UFAO	187.64 ± 6.2a	16.67 ± 0.14b	11.21 ± 0.12c	7.29 ± 0.05c	12.04 ± 1.27bc
72	1037	α-Ocimene ^J^	93	2		679.06 ± 22.5a	60.04 ± 7.26b	26.77 ± 2.07c	NF	27.13 ± 0.64c
73	1191	Limonene ^W,J^	68	1		353.41 ± 27.2a	28.49 ± 1.04b	20.09 ± 0.85b	10.04 ± 0.66b	16.8 ± 3.05b
74	1353	Rose oxide ^W,J^	139	2		26.9 ± 1.25a	9.4 ± 0.32b	4.88 ± 0.08cd	4.01 ± 0.03d	5.31 ± 0.3c
75	1724	Lilac alcohol ^W^		2		NF	5.54 ± 0.15a	3.87 ± 0.2c	4.01 ± 0.01bc	4.13 ± 0.03b
76	1473	Cosmene ^J^		2		NF	NF	9.01 ± 0.19a	NF	NF
77		Geranial ^N^		2		83.01 ± 2.27a	16.54 ± 1.38b	10.58 ± 0.24c	NF	8.47 ± 0.51c
Ketones										
78	1167	2,6-Dimethyl-4-heptanone ^W,J^	57	2	UFAO	52.15 ± 2.42a	8.06 ± 0.58d	15.45 ± 1.56c	22.78 ± 1.73b	14.43 ± 0.29c
79	1416	3-Octen-2-one ^J^	55	2	UFAO	NF	7.95 ± 0.5c	3.16 ± 0.13cd	31.07 ± 1.09a	24.49 ± 7.54b
80	955	2,3-Butanedione ^W,J^	43	1	MR	NF	32.56 ± 2.15c	22.02 ± 0.36d	77.8 ± 6.88a	61.51 ± 10.39b
81	1337	Sulcatone ^W,J^	43	1	CR	24.55 ± 0.4b	46.22 ± 1.46a	22.15 ± 0.2c	14.09 ± 1.86d	21.21 ± 0.7c
82	1596	6-Methyl-3,5-heptadiene-2-one ^B,W,J^	43	2	CR	5.47 ± 0.44c	47.45 ± 1.77a	11.86 ± 0.61c	12.58 ± 2.38c	30.45 ± 14.16b
83	1289	Acetoin ^W,J^	45	1		NF	265.02 ± 10.57c	76.37 ± 8.53d	581.23 ± 4.88a	361.45 ± 9.94b
84	1656	Acetophenone ^B,W,J^	105	2		26.27 ± 0.49a	5.66 ± 0.17c	3.65 ± 0.02d	5.15 ± 0.46c	8.45 ± 0.17b
Furans										
85	1224	2-Pentyl furan ^W,J^	81	2	UFAO	NF	NF	6.89 ± 0.45c	77.63 ± 1.2a	20.7 ± 2.06b
86	1469	Furfural ^B,W,J^	96	1	MR	NF	58.72 ± 4.07c	39.49 ± 7.05c	339.24 ± 17.64a	234.73 ± 14.13b
87	1509	2-Acetylfuran ^B,W,J^	95	2	MR	NF	NF	4.23 ± 0.14c	19.52 ± 0.56a	18.06 ± 1.6b
88	1578	5-Methyl-2-furfural ^B,W,J^	110	1	MR	NF	3.65 ± 0.29c	2.69 ± 0.23c	9 ± 0.85b	13.06 ± 1.44a
89	1270	5-Hydroxymethyl-2-furaldehyde ^J^	97	2	MR	NF	NF	14.13 ± 5.97b	7.34 ± 0.93b	18.8 ± 6.68a
Benzenes										
90	1259	Styrene ^J^	104	2		130.9 ± 5.37a	22.31 ± 2.14b	19.53 ± 0.4b	19.56 ± 0.17b	19.75 ± 0.21b
91	1281	o-Cymene ^J^	119	2		94.21 ± 3.59a	7.68 ± 0.12b	7.53 ± 0.32b	4.44 ± 0.34bc	5.73 ± 0.2b
92	1022	Toluene ^J^	91	2		38.46 ± 1.4a	4.29 ± 0.86b	4.4 ± 0.25b	4.78 ± 0.32b	4.72 ± 0.18b
93	1757	Naphthalene ^W,J^	128	1		16.47 ± 0.65a	3.25 ± 0.12b	2.28 ± 0.1c	2.47 ± 0.03c	2.52 ± 0.03c
94	1895	2-Methyl naphthalene ^W,J^	142	2		15.68 ± 0.54a	3.24 ± 0.11b	2.47 ± 0.07c	NF	NF
Pyrazines										
95	1385	2-Ethyl-6-methyl, pyrazine ^W,J^	121	1	MR	29.19 ± 0.97a	1.09 ± 0.09c	1.65 ± 0.04c	4.87 ± 0.43b	1.58 ± 0.4c
96	1435	2,6-Diethyl, pyrazine ^W,J^	135	1	MR	NF	3.56 ± 0.47b	1.35 ± 0.09c	9.76 ± 1.14a	2.52 ± 0.39c
97	1462	5-Ethyl-2,3-dimethylpyrazine ^W,J^	135	1	MR	NF	NF	1.57 ± 0.05bc	7.18 ± 3.13a	2.56 ± 0.31b
Phenols										
98	2010	Phenol ^W,J^	95	1		5.16 ± 0.17a	NF	1.06 ± 0.01c	1.29 ± 0.1b	0.85 ± 0.04d
99	2146	4-ethenyl-2-methoxyphenol J	135	2		1.81 ± 0.38a	NF	NF	NF	NF

Reported by ^B^: reported by Buttery et al. [22] for the study of Thompson Seedless raisins, ^J^: reported by Joulain and Fourniol [21] for the study of California sun-dried raisins, ^W^: reported by Wang et al. [2,3] for different raisin varieties in air-dried and sun-dried, ^N^: never reported as volatile compounds of raisins. Mean ± standard deviation (*n* = 3) of the same compounds followed by different letters are significantly different (*p* < 0.05), the free volatiles were compared separately. The characteristic ion (*m*/*z*) was used for choosing the corresponding compound in order to avoid possible interference by other compounds. Identification method: 1, identified, mass spectrum and retention indices (RI) were in accordance with standards; 2, tentatively identified, mass spectrum matched in the standard NIST 2008 library and RI matched with the National Institute of Standards and Technology (NIST) Standard Reference Database (NIST Chemistry WebBook); 3, tentatively identified, mass spectrum agreed with the standard NIST 2008. PAD (Pre-treated air-dried); SD (Sun-dried); PSD (Pre-treated sun-dried). UFAO: unsaturated fatty acid oxidation; MR: Maillard reaction.

**Table 2:** Odour activity values of the 30 most potent volatiles in the fresh and dried grapes.

S/N (Serial Number)	Compound Name	OTV	Aroma Descriptor	Aromatic Series	OAV				
					Fresh	AD	PAD	SD	PSD
1	Pentanal	12	Fat, Green ^e^	Green	13.99	5.78	5.35	0	6.75
2	Hexanal	4.5	Green ^e^	Green	1549.49	81.21	63.54	121.69	122.46
3	(*E*)-2-Hexenal	17	Green ^e^	Green	194.37	3.75	7.92	1.05	2.65
4	(*E,E*)-2,4-Nonadienal	0.09	Green ^b^	Green	1996	2681	1060	1186	1048
5	Geranic acid	40	Green ^e^	Green	12.23	3.73	2.15	0.17	0.64
6	Octanal	0.7	Honey, Green ^e^	Green	552.64	0	0	0	0
7	Nonanal	1	Green, Fruity ^e^	Green; Fruity	6.38	1.18	1.38	1.74	1.49
8	Decanal	0.1	Sweet, citrus, green ^d^	Green; Fruity	0	0	18.76	18.4	20.78
9	(*Z*)-3-Hexen-1-ol	100	Fruity, green ^b^	Green; Fruity	3.87	0.52	0.39	0	0.32
10	2-Pentyl furan	6	Fruity, green, sweet ^e^	Green; Fruity	0	0	1.15	12.94	3.45
11	(*E*)-2-Octenal	3	Green, fatty, nut ^e^	Green; Fatty	7.57	8.9	1.56	3.8	2.79
12	(*E*)-2-Nonenal	0.08	Green, fat ^a^	Green; Fatty	0	0	31.29	92.99	0
13	(*E*)-2-Heptenal	13	Fatty, soapy, tallow ^e^	Fatty	2.11	8.56	1.42	4.45	1.91
14	(*E,E*)-2,4-Heptadienal	49	Fatty, hay ^b^	Fatty	0	0	9.48	1.77	2.27
15	Ethyl hexanoate	1	Fruity, apple-like ^a, e^	Fruity	10.63	14.38	11.67	0	3.16
16	Ethyl hexadecanoate	1	Fruity, apple-like ^a, e^	Fruity	47.55	11.39	7.71	8.13	8.29
17	Linalool	6	Fruity, sweet, grape ^b, e^	Fruity	41.2	2.57	2.27	0.8	0.82
18	Limonene	10	Citrus-like	Fruity	35.34	2.85	2.01	1	1.68
19	Geranial/ α-Citral	32	lemon, mint flvt	Fruity	2.59	0.52	0.33	0	0.26
20	β-damascenone	0.09	Sweet, floral, fruity ^e^	Fruity; Floral	699.21	36.17	35.64	24.54	25.15
21	Geraniol	40	Floral, rose, citrus ^e^	Fruity; Floral	5.88	1.79	0.54	0.04	0.64
22	Phenylacetaldehyde	4	Flowery, Rose ^e^	Floral	96.74	81.67	40.85	25.47	0
23	α-Terpineol	350	Floral, sweet ^e^	Floral	1.08	0.1	0.05	0	0.06
24	β-Ionone	0.007	Balsamic, rose ^e^	Floral	772.31	152.95	107.55	0	0
25	Rose oxide	0.5	Rose, floral ^a, e^	Floral	53.79	18.8	9.76	8.02	10.62
26	2,6-Diethyl, pyrazine	6	Roasted, nutty ^b^	Roasted	0	0.59	0.22	1.63	0.42
27	5-Ethyl-2,3-dimethylpyrazine	3	Nutty roasted, woody ^e^	Roasted	0	0	0.52	2.39	0.85
28	Heptanal	3	Dry fish, solvent, smoky ^e^	Chemical	28.37	11.41	8.25	16.06	12.22
29	3-Methyl-1-butanol	300	Malt, whiskey ^a^	Chemical	0	9.38	4.85	3.77	4.96
30	Acetic acid	60,000	Vinegar ^c^	Chemical	2.25	4.43	3.01	3.48	1.9

Aroma descriptors were obtained from “Flavornet and human odor space” (^a^ http://www.flavornet.org/flavornet.html (accessed on 3 March 2021)), the LRI and odor database (^b^ http://www.odour.org.uk/odour/index.html (accessed on 3 March 2021)) and from the reported literature (^c^ Qian & Wang, [39]; ^d^ Wang et al. [3]; ^e^ Wu et al. [19]). OTV (odor threshold value); OAV (odor active value); AD (air-dried); PAD (pre-treated air-dried); SD (sun-dried); PSD (pre-treated sun-dried).

## Data Availability

Not applicable.

## Supplementary Materials

The following are available online at https://www.mdpi.com/2304-8158/10/3/559/s1, Figure S1: The effect of drying treatments on the changing of TSS during the drying of centennial Seedless raisins, Table S1: The concentration of free- and glycosidically bound VOCs in fresh and dried centennial Seedless grapes, File S1: List of chemicals.
