# Supplementary material for: Drying Treatments Change the Composition of Aromatic Compounds from Fresh to Dried Centennial Seedless Grapes
Source: Foods. 2021 Mar 8;10(3):559. doi: 10.3390/foods10030559 (PMC7998674; doi:10.3390/foods10030559)
Supplement: Supplementary file 1 [file foods-10-00559-s001.pdf]

File S1: List of chemicals.

2.2. Chemicals

The sodium hydroxide (NaOH), glucose, sodium chloride (NaCl), sodium dihydrogen phosphate, and citric acid were obtained from Beijing Chemical Works (China). The chemicals, dichloromethane, methanol, and ethanol that were used for the extraction of VOCs were purchased by Honeywell (Morris Township, NJ®). The distilled water was taken from a Milli-Q purification system (Millipore, Bedford, MA).

For the identification and quantification of free volatile compounds, the following standards were purchased from Sigma®–Aldrich (St. Louis, MO): 1-octanol (99.0%), 3-methyl-1-butanol (99.0%), 2-ethyl-1-hexanol (99.0%), 1-octen-3-ol (98.0%), 1-nonanol (99.0%), octanoic acid (99.0%), benzyl alcohol (98.0%), 2-methyl-propanoic acid (99.5%), heptanoic acid (99.0%), hexanoic acid (99.5%), hexanal (98.0%), (E)-2-hexenal (98.0%), nonanal (95.0%), octanal (99.0%), benzaldehyde (99.0%), ethyl acetate (99.8%), diethyl succinate (99.0%), benzene acetaldehyde (90.0%), ethyl phenylacetate (99.0%), ethyl nonanoate (98.0%), methyl salicylate (99.0%), acetoin (96.0%), 6-methyl-5-hepten-2- one (99.0%), α-terpineol (90.0%), ϱ-cymene (98.0%), limonene (97.0%), neral (95.0%), hotrienol (97.0%), terpinolene (97.0%), β-damascenone (> 90.0%), linalool (97.0%), β-citronellol (95.0%), geranyl acetone (35% nerylacetone), geraniol (99.5%), geranic acid (85.0%), 2,3-diethylpyrazine (98.0%), 5-methyl-2-furfural (99.0%), 2-ethyl-6-methyl, 4-methyl-2-pentanol (98.0%, internal standard), 3-ethyl-2,5-dimethyl pyrazine (98.0%), pyrazine (99.5%), naphthalene (99.0%), phenol (99.9%) and furfural (99.5%).

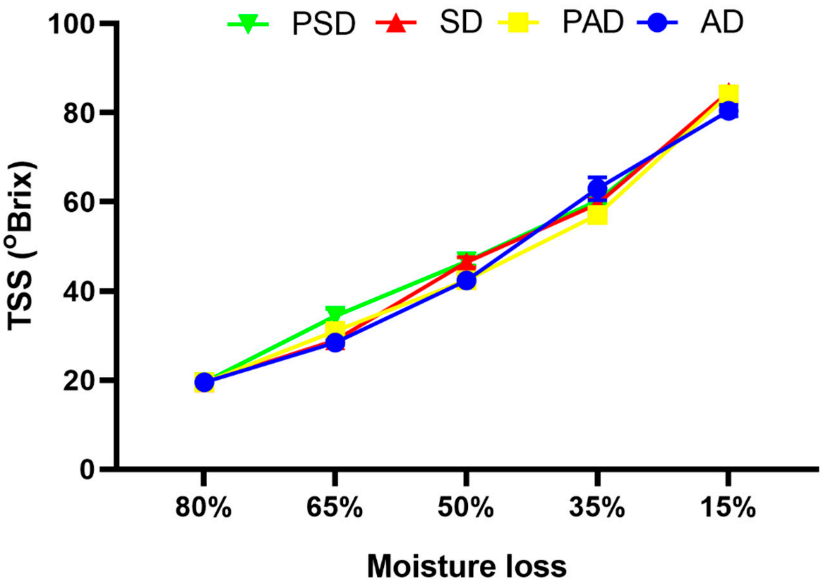

Figure 1. The effect of drying treatments on the changing of TSS during the drying of centennial Seedless raisins.

Table 1. The concentration of free- and glycosidically bound VOCs in fresh and dried centennial Seedless grapes.

	RI	Compound Name	Ion *m/z*	ID	Source			Fresh	AD-Raisin	PAD-Raisin	SD-Raisin	PSD-Raisin	Fresh	AD-Raisin	PAD-Raisin	SD-Raisin	PSD-Raisin
								Free-form Volatile compounds					Bound-form Volatile compounds				
1	975	Pentanal [W,J]	44	2	UFAO	12 [3,7]	Fat, Green [g]	167.92 ± 13.06a	69.36 ± 2.34cd	64.23 ± 2.51d	86.8 ± 6.14bc	81.01 ± 7.64b	574.93 ± 6.1	NF	NF	102.67 ± 3.5	105.7 ± 2.38
2	1066	Hexanal [B,W,J]	44	1	UFAO	4,5 [3,7]	Green [g]	6972.72 ± 365.7a	365.44 ± 24.86b	285.95 ± 2.96b	547.62 ± 13.12b	551.07 ± 5.42b	641.1 ± 6.64	25.21 ± 0.3	22.57 ± 1.62	103.51 ± 0.6	115.36 ± 3.1
3	1178	Heptanal [B,W,J]	44	2	UFAO	3 [3,7]	Dry fish, solvent, smoky [g]	86.1 ± 7.32a	34.22 ± 0.87bc	24.76 ± 1.43c	48.17 ± 3.71b	36.66 ± 0.74bc	NF	NF	NF	NF	NF
4	1292	Octanal [B,W]	43	2	UFAO	0.7 [3,7]	Honey, Green, Fatty [g]	386.85 ± 45.07a	NF	NF	NF	NF	199.36 ± 27.37	NF	NF	NF	NF
5	1393	Nonanal [B,W,J]	57	1	UFAO	1 [3,7]	Green, Fruity [g]	6.38 ± 0.08a	1.18 ± 0.23c	1.38 ± 0.07bc	1.74 ± 0.35b	1.49 ± 0.27bc	0.51 ± 0.06	0.86 ± 0.01	0.69 ± 0	0.06 ± 0	0.15 ± 0.01
6	1501	Decanal [B,W,J]	43	1	UFAO	0.1 [3]	Sweet, citrus, green [e]	NF	NF	1.88 ± 0.03a	1.84 ± 0.07a	2.08 ± 0.44a	NF	NF	NF	0.47 ± 0.04	0.9 ± 0.08
7	1217	*(E)*-2-Hexenal [B,W,J]	41	1	UFAO	17 [3,7]	Green [g]	3304.21 ± 85.45a	63.7 ± 3.77c	134.68 ± 9.12b	17.77 ± 0.32c	44.97 ± 2.29c	152.77 ± 23.02	2.06 ± 0.32	3.79 ± 0.45	NF	NF
8	1325	*(E)*-2-Heptenal [B,W,J]	41	2	UFAO	13 [3,7]	Fatty, soapy, tallow [g]	27.41 ± 2.29c	111.29 ± 6.05a	18.41 ± 0.37c	57.83 ± 9.67b	24.88 ± 3.41c	380.58 ± 18.83	NF	NF	59.76 ± 0.92	61.63 ± 0.94
9	1434	*(E)*-2-Octenal [B,W,J]	41	2	UFAO	3 [3]	Green, fatty, nut [g]	22.7 ± 0.75a	6.69 ± 1.41d	4.68 ± 0.16e	11.39 ± 1.92b	8.38 ± 0.92c	NF	NF	NF	NF	NF
10	1539	*(E)*-2-Nonenal [B,W]	43	2	UFAO			NF	NF	2.5 ± 0.1b	7.44 ± 0.38a	NF	NF	30.06 ± 0.75	33.12 ± 1.31	NF	8.24 ± 0.72
11	1497	*(E,E)*-2,4-Heptadienal [B,W,J]	81	2	UFAO	49 [4]	Fatty, hay [b]	NF	NF	NF	86.71 ± 4.17b	111.18 ± 7.03a	NF	NF	NF	26.27 ± 0.84	42.16 ± 1.52
12	1705	*(E,E)*-2,4-Nonadienal [B,W,J]	81	2	UFAO	0.09 [3]	Green	179.65 ± 3.2b	241.35 ± 13.59a	95.46 ± 8c	106.78 ± 12.96c	94.33 ± 4.12c	NF	NF	NF	NF	NF
13	1530	Benzaldehyde [B,W,J]	77	1	MR	350 [3,7]	Sweet, fruity [g]	13.43 ± 0.13b	25.6 ± 1.57a	15.2 ± 0.64b	14 ± 1.75b	14.05 ± 1.46b	7.9 ± 1.16	0.72 ± 0.03	1.64 ± 0.14	5.82 ± 0.11	2.48 ± 0.1
14	1650	Phenylacetaldehyde [B,W,J]	91	1	MR	4 [3,7]	Flowery, Rose [g]	386.96 ± 11.48a	326.67 ± 21.3b	163.4 ± 23.81c	141.21 ± 11.87d	101.89 ± 9.17e	8.22 ± 1.76	6.05 ± 0.36	7.32 ± 0.25	2.11 ± 0.22	4.87 ± 0.37
<u>Alcohol</u>								-									
15	1205	1-Pentanol [W,J]	52	2	UFAO	4000 [3]	Balsamic, almond [c]	14.81 ± 0.74a	8.54 ± 0.77c	8.69 ± 0.28c	11.92 ± 0.66b	12.99 ± 0.43b	16.85 ± 2.23	2.45 ± 0.09	6.73 ± 1.11	4.45 ± 0.12	8.51 ± 0.28
16	1349	1-Hexanol [W,J]	56	2	UFAO	2500 [3]	green [b]	901.25 ± 76.68a	74.36 ± 4.56c	156.45 ± 7.01b	110.57 ± 11.26bc	128.71 ± 2.79bc	59.48 ± 0.34	17.33 ± 1.1	73.78 ± 3.68	34.19 ± 0.42	79.9 ± 2.56
17	1453	1-Heptanol [W,J]	70	2	UFAO	1000 [3,5]	Grape, sweet [c]	9.65 ± 0.22a	NF	2.21 ± 0.01c	7.13 ± 0.95b	7.58 ± 0.83b	19.73 ± 0.89	1.13 ± 0.02	3.68 ± 0.07	3.84 ± 0.01	4.24 ± 0.06
18	1555	1-Octanol [B,W,J]	56	1	UFAO	130 [2,3]	Citrus, rose [c]	6.71 ± 0.22a	2.64 ± 0.19c	1.37 ± 0.05d	2.63 ± 0.46c	5.55 ± 0.26b	2.45 ± 0.42	0.78 ± 0.04	1.84 ± 0.15	0.74 ± 0.02	1.55 ± 0.23
19	1657	1-Nonanol [W,J]	56	1	UFAO	50 [3]	Floral [b]	1.93 ± 0.06a	0.58 ± 0.01bc	0.32 ± 0.01d	0.51 ± 0.06c	0.7 ± 0.12b	0.36 ± 0.01	0.29 ± 0.01	0.39 ± 0.02	0.11 ± 0.01	0.11 ± 0
20	1411	2-Octanol [B,W,J]	45	2	UFAO	NF	NF	0.83 ± 0.06a	NF	NF	NF	NF	1.39 ± 0.17	0.04 ± 0	0.26 ± 0.02	0.29 ± 0.01	0.33 ± 0.02

21	1488	2-Nonanol [J]	45	2	UFAO	58 [4]	Fruity, green [d, f]	1.86 ± 0.07a	NF	NF	NF	NF	NF	NF	NF	NF	NF
22	1449	1-Octen-3-ol [B,W,J]	57	1	UFAO	3 [3,7]	Mushroom, fruity [b, g]	14.93 ± 0.62d	23.18 ± 3.3c	24.99 ± 0.8c	36.48 ± 5.83b	52.42 ± 2.3a	2.33 ± 0.06	0.22 ± 0.02	0.71 ± 0.06	0.98 ± 0.01	1.46 ± 0.13
23	1614	(E)-2-Octen-1-ol [W,J]	57	2	UFAO	18-5	Fatty, rancid [b]	3.15 ± 0.07d	10.86 ± 0.09a	5.65 ± 0.34c	2.5 ± 0.57e	7.42 ± 0.34b	3.17 ± 0.23	0.1 ± 0.01	0.31 ± 0.01	0.87 ± 0.03	0.93 ± 0.05
24	1487	2-Ethyl-1-hexanol [W,J]	57	1	UFAO	270000 [3]	Floral, sweet fruity [c]	15.26 ± 0.41a	0.78 ± 0.08c	3.31 ± 0.01b	1.03 ± 0.51c	3.24 ± 0.15b	6.45 ± 0.22	1.15 ± 0.12	1.78 ± 0.3	4.57 ± 0.05	5.13 ± 0.77
25	1395	(Z)-3-Hexen-1-ol [J]	67	2	UFAO	100 [3,5]	Fruity, green [b]	387.23 ± 28.45a	52.3 ± 0.71b	38.83 ± 0.74b	NF	32.11 ± 0.19b	69.32 ± 8.72	11.61 ± 0.59	14.74 ± 1.14	8.59 ± 0.29	11.54 ± 1.86
26	1203	3-Methyl-1-butanol [W,J]	55	1	UFAO	300 [3]	Malt, whiskey [a]	NF	1814.98 ± 32.3a	1455.15 ± 26.93b	1130.05 ± 32.01c	1487.78 ± 24.13b	2719.5 ± 278.12	458.24 ± 30.31	797.17 ± 60.47	326.35 ± 3.34	543.99 ± 27.7
27	1458	Sulcatol [J]	95	2	CR	2000 [3]	Fruity, sweet [g]	9.84 ± 0.14a	2.73 ± 0.04b	1.68 ± 0.03c	0.74 ± 0.23d	1.44 ± 0.16c	2.41 ± 0.26	0.55 ± 0.04	1.64 ± 0.24	0.47 ± 0.02	1.84 ± 0.09
28	942	Ethyl alcohol [J]	31	2		100000 [3]	Sweet [a]	13143.64 ± 1679ab	13526.01 ± 531a	7751.14 ± 85.13c	8197.47 ± 165c	11920.23 ± 732d	228.96 ± 6.79	NF	NF	81.83 ± 1.42	25.35 ± 4.07
29	1317	3-Methyl-2-buten-1-ol [W,J]	71	2		NF	Fruity	NF	NF	NF	NF	NF	2.28 ± 0.12	0.65 ± 0.03	0.87 ± 0.12	0.41 ± 0.01	1.04 ± 0.04
30	1879	Benzyl alcohol [W,J]	79	1		10000 [3,7]	Fruity, sweet [g]	279.02 ± 18.52a	283.38 ± 16.54a	109.02 ± 3.61b	23.96 ± 0.05c	120.75 ± 5.05b	348.41 ± 11.83	22.8 ± 0.77	327.56 ± 17.44	103.82 ± 4.8	619.68 ± 19.48
31	1914	Phenylethyl alcohol [W,J]	91	2		1100 [3]	Floral, Rose, Honey [g]	363.21 ± 32.76a	358.05 ± 37.8a	92.78 ± 2.82c	43.41 ± 6.67d	155.29 ± 13.89b	426.82 ± 17.4	10.54 ± 1.39	66.62 ± 3.77	71.56 ± 0.43	97.49 ± 1.15
32	1148	1-Butanol [J]	56	2		500 [3]	Fruity, floral [b]	248.09 ± 35.63a	47.43 ± 1.51b	44.66 ± 0.93b	37.55 ± 4.11b	32.58 ± 2.4b	NF	40.81 ± 0.03	34.19 ± 1.86	11.81 ± 0.11	29.64 ± 0.67
<u>Ester</u>								-				-					
33	1227	Ethyl hexanoate [W,J]	88	1	UFAO	1 [3,7]	Fruity, apple like [a, g]	10.63 ± 2.55b	14.38 ± 4.31a	11.67 ± 0.21b	6.32 ± 2.87	3.82 ± 1.48d	NF	NF	NF	NF	NF
34	1570	Ethyl octanoate [W,J]	88	1	UFAO	377 [3]	Fruity, floral [b]	5.56 ± 0.45a	2.43 ± 0.21b	1.13 ± 0.03d	1.17 ± 0.07d	1.72 ± 0.13c	NF	0.92 ± 0.04	0.73 ± 0.01	NF	NF
35	1378	Methyl octanoate [J]	74	2	UFAO	200 [3]	Fruity, citrus like [a]	NF	NF	0.14 ± 0b	0.14 ± 0.01b	0.17 ± 0.02a	NF	NF	NF	NF	NF
36	1570	Ethyl nonanoate [J]	88	1	UFAO	377 [3]	Fruity, floral [b]	NF	NF	0.16 ± 0a	NF	NF	NF	NF	NF	NF	NF
37	1227	Ethyl hexadecanoate [W,J]	88	1	UFAO	1 [3,7]	Fruity, apple like [a, g]	47.55 ± 5.31a	11.39 ± 0.54b	7.71 ± 0.28b	8.13 ± 0.09b	8.29 ± 0.09b	NF	NF	NF	NF	NF
38	2163	Methyl hexadecanoate [J]	74	2	UFAO	NF	NF	0.44 ± 0.06a	0.14 ± 0.01b	0.07 ± 0c	NF	NF	2.36 ± 0.05	NF	NF	NF	NF
39	1636	Butyrolactone [W,J]	42	2	UFAO	NF	NF	3.75 ± 0.5d	8.68 ± 0.12a	6.58 ± 0.52b	5.57 ± 0.4c	8.19 ± 0.74a	NF	NF	NF	NF	NF
40	2035	γ-Nonalactone [W,J]	85	2	UFAO	30-6	Coconut, peach [a]	3.34 ± 0.29b	8.99 ± 0.68a	1.49 ± 0.04c	1.43 ± 0.12c	1.81 ± 0.57c	0.84 ± 0.11	0.6 ± 0.02	0.8 ± 0.07	0.17 ± 0	0.26 ± 0.01
41	885	Ethyl acetate [W,J]	43	1		5000 [3,7]	Fruity, solvent [g]	322.94 ± 20.33d	1509.48 ± 34.63a	1400.73 ± 63.66b	368.82 ± 16.62d	801.43 ± 22.32c	113.29 ± 2.4	9.26 ± 0.36	11.58 ± 8.66	20.61 ± 0.26	25.77 ± 3.7
42	1639	Ethyl decanoate [W]	88	1		200-4	Fruity, apple [b]	NF	NF	NF	NF	NF	NF	8.79 ± 0.04	12.17 ± 8.39	NF	NF

43	1677	Diethyl succinate [W,J]	101	1		500000 [3]	Fruity, wine [a, f]	NF	NF	1.57 ± 0.04a	NF	1.46 ± 0.06b	NF	NF	NF	NF	NF
44	1782	Methyl salicylate JF	120	1		40 [3]	Green Pine [a, g]	NF	NF	2.98 ± 0.09a	NF	NF	3.62 ± 0.42	3.81 ± 0.13	2.95 ± 0.29	0.65 ± 0.01	0.7 ± 0
45	1253	Ethyl salicylate [J]		2		NF	Green, mint [a]	NF	NF	0.54 ± 0.01a	0.36 ± 0.01b	NF	NF	NF	NF	NF	0.58 ± 0.02
46	942	Ethyl alcohol [J]	31	2		100000 [3]	Sweet [a]	13143.64 ± 1679.41ab	13526.01 ± 531.25a	7751.14 ± 85.13c	8197.47 ± 165.51c	11920.23 ± 732.92b	NF	NF	NF	NF	NF
47	1470	β-Ionone [J]	177	2	CR	0.007 [3,7]	Balsamic, rose [g]	5.41 ± 0.21a	1.07 ± 0.05b	0.75 ± 0.02c	NF	NF	24.28 ± 0.94	0.84 ± 0.08	1.74 ± 0.03	3.74 ± 0.06	3.81 ± 0.04
Acid																	
48	1740	Pentanoic acid [W,J]	60	2	UFAO	3000 [3]	Sweet	- NF	NF	74.41 ± 2.76b	187.82 ± 8.88a	- NF	334.16 ± 19.81	NF	NF	64.84 ± 0.62	71.05 ± 0.65
49	1847	Hexanoic acid [B,W,J]	60	1	UFAO	3000 [3,7]	Raincid, Chees, Fatty [g]	375.48 ± 19.8a	153.07 ± 13.89b	161.91 ± 26.48b	214.5 ± 11.34b	161.37 ± 56.99b	NF	NF	NF	NF	NF
50	1953	Heptanoic acid [B,W,J]	60	1	UFAO	3000 [3]	Sweety, cheesy [f]	3.19 ± 0.57a	2.58 ± 0.17ab	2.17 ± 0.11b	2.52 ± 0.35ab	2.96 ± 0.66a	309.89 ± 7.8	NF	48.86 ± 0.79	49.38 ± 0.49	52.39 ± 2.11
51	2060	Octanoic acid [B,W,J]	60	1	UFAO	3000 [3,7]	Raincid, Chees, Fatty [g]	2.41 ± 0.16b	4.63 ± 0.19a	2.33 ± 0.22b	2.29 ± 0.12b	2.11 ± 0.72b	315.84 ± 12.26	NF	49.66 ± 0.25	49.72 ± 0.58	52.51 ± 2.02
52	2166	Nonanoic acid [B,W,J]	60	2		3000 [3]	Green, fat [a, f]	20.16 ± 1.9a	4.45 ± 0.17c	8.69 ± 1.26b	3.53 ± 0.03c	4.76 ± 0.24c	577.3 ± 22.3	NF	88.35 ± 4.22	90.96 ± 0.77	91.17 ± 1.08
53	2484	Dodecanoic acid [W,J]	73	2	UFAO	10000 [3]	Dry, metallic [f]	NF	NF	10.9 ± 2.72a	NF	NF	NF	NF	NF	NF	NF
54	1950	2-Ethylhexanoic acid [W,J]	88	1		NF	NF	NF	NF	28.97 ± 0.51a	NF	NF	NF	NF	NF	NF	NF
55	1433	Acetic acid [J]		2		60000 [5]	Vinegar [d]	135518.4 ± 11152.8d	265800.8 ± 12361.5a	180758.57 ± 7545.9c	209013.96 ± 17456.5b	114244.24 ± 6693.1e	3371.18 ± 53.85	27031.11 ± 1012.09	22028.15 ± 1192.06	5804.92 ± 139.71	2457.09 ± 181.71
Terpenes																	
56	1011	α-Terpinene [N]	121	2				- 377.96 ± 11.76a	36.38 ± 3.67b	18.52 ± 0.64c	NF	19.33 ± 2.3c	45.26 ± 1.15	14.31 ± 0.7	14.1 ± 2.97	6.57 ± 0.03	8.15 ± 0.37
57	1286	Terpinolene [J]	93	1		200 [3]	Piney [g]	185.51 ± 8.78a	17.59 ± 0.51b	14.86 ± 0.16bc	9.47 ± 0.09c	NF	34 ± 4.32	10.66 ± 0.3	8.76 ± 1.56	6.33 ± 0.08	7.28 ± 0.04
58	1473	Nerol oxide [W,J]	68	2		3000 [7]	Oil, flowery [g]	141.28 ± 6.83b	226.12 ± 6.18a	65.67 ± 0.56d	22.77 ± 4.76e	99.44 ± 6.57c	47.94 ± 1.52	20.94 ± 2.1	29.27 ± 1.6	12.81 ± 0.53	34.76 ± 1.44
59	1548	Linalool [W,J]	71	1		6 [3,6]	Fruity, sweet, grape [b, e]	247.23 ± 32.98a	15.44 ± 0.71b	13.63 ± 0.49b	4.81 ± 0.39b	4.9 ± 0.69b	14.15 ± 0.02	1.58 ± 0.2	6.42 ± 0.7	2.3 ± 0.15	4.84 ± 0.3
60	1610	Hotrienol [W,J]	71	1		110 [7]	Fresh, floral, fruity [g]	99.66 ± 2.9a	31.05 ± 0.44b	25 ± 0.43c	14.51 ± 0.82d	10.67 ± 0.16e	2.88 ± 0.04	0.15 ± 0.2	0.58 ± 0.01	0.46 ± 0	0.61 ± 0.05
61	1620	p-Menth-1-en-9-al [W,J]	94	2		NF	NF	40.38 ± 0.5a	11.18 ± 0.09b	6.55 ± 0.12c	6.36 ± 0.32cd	5.77 ± 0.43c	27.42 ± 1.03	4.98 ± 0.01	4.68 ± 0.06	4.35 ± 0.06	4.68 ± 0.01
62	1698	α-terpineol [B,W,J]	59	1		350 [3]	Floral, sweet [g]	77.53 ± 2.74a	12.66 ± 0.6b	9.63 ± 0.13c	6.36 ± 0.15d	7.29 ± 0.45d	30.37 ± 0.32	6.92 ± 0.46	12.76 ± 0.73	5.48 ± 0.13	7.82 ± 0.07
63	1739	cis-Pyran linalool oxide [W,J]		2		NF	Floral, fruity, Citrus [a]	8.69 ± 0.31a	6.28 ± 0.08b	2.86 ± 0.08c	2.69 ± 0.07c	2.45 ± 0.23c	5.74 ± 0.26	1.3 ± 0.06	2.25 ± 0.04	2.59 ± 0.02	5.83 ± 0.27

64	1764	β-Citronellol [J]	69	1		62 [5]	Sweet, citrus like [f]	7.62 ± 0.28a	2.84 ± 0.15b	1.79 ± 0.45c	NF	NF	3.77 ± 0.3	0.4 ± 0.03	0.94 ± 0.02	0.76 ± 0	1.81 ± 0.09
65	1799	Nerol [W,J]	69	2		300 [3,7]	Flower, grass, floral [g]	276.43 ± 3.01a	74.14 ± 3.33b	26.37 ± 0.15d	3.22 ± 0.18e	30.93 ± 2.77c	25.52 ± 1.77	11.59 ± 1.1	26.55 ± 1.36	3.63 ± 0.14	12.53 ± 0.82
66	1825	β-damascenone [W,J]	69	1		0.09 [6]	Sweet, floral, fruity [g]	62.93 ± 7.23a	3.26 ± 0.13b	3.21 ± 0.1b	2.21 ± 0.07b	2.26 ± 0.4b	372.36 ± 6.9	14.72 ± 1.6	1.21 ± 0.12	62.88 ± 0.66	276.17 ± 9.99
67	1847	Geraniol [W,J]	69	1		40 [3,7]	Floral, rose, citrus [g]	235.23 ± 2.38a	71.45 ± 6.14b	21.77 ± 0.09c	1.54 ± 0.15d	25.63 ± 2.38c	12.78 ± 1.55	7.64 ± 1.19	20.8 ± 2.39	2.32 ± 0.41	12.18 ± 0.96
68	2049	Nerolidol 2 [J]	41	2		NF	NF	NF	NF	0.58 ± 0a	NF	NF	NF	NF	NF	NF	NF
69	2340	Geranic acid [W,J]	69	1		40 [7]	Green [g]	489.07 ± 56.92a	149.32 ± 5.42b	86.18 ± 6.87c	16.74 ± 0.4d	25.57 ± 1.4d	956.52 ± 34.25	7.46 ± 0.36	44.8 ± 1.05	147.34 ± 1.76	162.6 ± 1.48
70	1857	Geranylacetone [B,W,J]	43	1	CR	60 [3]	Rose, floral [b, f]	6.1 ± 0.74a	1.4 ± 0.52b	0.8 ± 0.07b	0.77 ± 0.07b	1.18 ± 0.1b	17.71 ± 0.62	0.64 ± 0.02	1.48 ± 0.1	2.78 ± 0.02	3.06 ± 0.06
71	1132	3,4-Dimethyl-2,4,6-octatriene [J]	121	2	UFAO	NF	NF	187.64 ± 6.2a	16.67 ± 0.14b	11.21 ± 0.12c	7.29 ± 0.05c	12.04 ± 1.27bc	41.53 ± 1.38	11.34 ± 0.5	8.3 ± 0.32	6.45 ± 0.04	7.68 ± 0.07
72	1037	α-Ocimene [J]	93	2		NF	Fruity	679.06 ± 22.5a	60.04 ± 7.26b	26.77 ± 2.07c	NF	27.13 ± 0.64c	47.65 ± 1.69	13.16 ± 0.7	15.74 ± 7.22	7.33 ± 0.03	11.26 ± 0.5
73	1191	Limonene [W,J]	68	1		10-wang	Citrus-like	353.41 ± 27.2a	28.49 ± 1.04b	20.09 ± 0.85b	10.04 ± 0.66b	16.8 ± 3.05b	35.53 ± 3.15	13.26 ± 0.6	25.63 ± 0.86	6.89 ± 0.14	8.59 ± 0.21
74	1353	Rose oxide [W,J]	139	2		0.5 [3,7]	Rose, floral [a, g]	26.9 ± 1.25a	9.4 ± 0.32b	4.88 ± 0.08cd	4.01 ± 0.03d	5.31 ± 0.3c	26.35 ± 0.98	5.37 ± 0.24	4.31 ± 0.28	4.26 ± 0.03	4.54 ± 0.01
75	1724	Lilac alcohol [W]		2		NF	NF	NF	5.54 ± 0.15a	3.87 ± 0.2c	4.01 ± 0.01bc	4.13 ± 0.03b	NF	NF	NF	NF	NF
76	1473	Cosmene [J]		2		NF	NF	NF	NF	9.01 ± 0.19a	NF	NF	30.92 ± 0.27	NF	NF	6.79 ± 0.16	6.54 ± 0.19
77		**Geranial** [N]		2				83.01 ± 2.27a	16.54 ± 1.38b	10.58 ± 0.24c	NF	8.47 ± 0.51c	32.94 ± 1.4	15.13 ± 0.9	14.58 ± 0.86	2.88 ± 0	16.07 ± 1.59
Ketones								-									
78	1167	2,6-Dimethyl-4-heptanone [W,J]	57	2	UFAO	NF	NF	52.15 ± 2.42a	8.06 ± 0.58d	15.45 ± 1.56c	22.78 ± 1.73b	14.43 ± 0.29c	64.81 ± 1.08	29.92 ± 0.9	29.71 ± 1.53	8.34 ± 0.13	1NF.6
79	1416	3-Octen-2-one [J]	55	2	UFAO	NF	Green, fruity [b]	NF	7.95 ± 0.5c	3.16 ± 0.13cd	31.07 ± 1.09a	24.49 ± 7.54b	NF	NF	NF	NF	NF
80	955	2,3-Butanedione [W,J]	43	1	MR	100 [8]	Butter [a]	NF	32.56 ± 2.15c	22.02 ± 0.36d	77.8 ± 6.88a	61.51 ± 10.39b	0.25 ± 0.05	NF	NF	1.41 ± 0.22	9.23 ± 0.51
81	1337	Sulcatone W,J	43	1	CR	50 [3]	Sweet, fruity f	24.55 ± 0.4b	46.22 ± 1.46a	22.15 ± 0.2c	14.09 ± 1.86d	21.21 ± 0.7c	4.66 ± 0.14	0.75 ± 0.01	3.95 ± 4.96	5.15 ± 0.05	9.27 ± 3.99
82	1596	6-Methyl-3,5-heptadiene-2-one [B,W,J]	43	2	CR	380 [3]	Warm, spicy [b]	5.47 ± 0.44c	47.45 ± 1.77a	11.86 ± 0.61c	12.58 ± 2.38c	30.45 ± 14.16b	NF	NF	NF	NF	NF
83	1289	Acetoin [W,J]	45	1		800 [3]	Buttery, cream [a]	NF	265.02 ± 10.57c	76.37 ± 8.53d	581.23 ± 4.88a	361.45 ± 9.94b	90.62 ± 11.8	NF	NF	15.56 ± 0.05	16.71 ± 0.19
84	1656	Acetophenone [B,W,J]	105	2		65 [3]	Sweet, flowery [a]	26.27 ± 0.49a	5.66 ± 0.17c	3.65 ± 0.02d	5.15 ± 0.46c	8.45 ± 0.17b	5.61 ± 0.22	3.18 ± 0.06	5.94 ± 2.23	1.02 ± 0.01	1.62 ± 0.22
Furans								-									
85	1224	2-Pentyl furan [W,J]	81	2	UFAO	6 [3,7]	Fruity, green, sweet [g]	NF	NF	6.89 ± 0.45c	77.63 ± 1.2a	20.7 ± 2.06b	NF	NF	NF	NF	NF

86	1469	Furfural [B,W,J]	96	1	MR	3000[3]	Sweet, almond [b, c]	NF	58.72 ± 4.07c	39.49 ± 7.05c	339.24 ± 17.64a	234.73 ± 14.13b	4.25 ± 0.19	1.65 ± 0.05	3.27 ± 0.19	0.74 ± 0.02	1.73 ± 0.12
87	1509	2-Acetylfuran [B,W,J]	95	2	MR	10000[3]	Smoky, sweet [b]	NF	NF	4.23 ± 0.14c	19.52 ± 0.56a	18.06 ± 1.6b	4.26 ± 0.06	NF	NF	NF	NF
88	1578	5-Methyl-2-furfural [B,W,J]	110	1	MR	NF	Warm, burnt, spicy [b]	NF	3.65 ± 0.29c	2.69 ± 0.23c	9 ± 0.85b	13.06 ± 1.44a	NF	NF	NF	NF	NF
89	1270	5-Hydroxymethyl-2-furaldehyde [J]	97	2	MR	NF	NF	NF	NF	14.13 ± 5.97b	7.34 ± 0.93b	18.8 ± 6.68a	NF	NF	NF	NF	NF
Benzenes								-									
90	1259	Styrene [J]	104	2		730[3]	Balsamic, gasoline [a]	130.9 ± 5.37a	22.31 ± 2.14b	19.53 ± 0.4b	19.56 ± 0.17b	19.75 ± 0.21b	0.36 ± 0.01	25.13 ± 0.8	18.34 ± 1.04	0.08 ± 0	0.11 ± 0.01
91	1281	o-Cymene [J]	119	2		NF	NF	94.21 ± 3.59a	7.68 ± 0.12b	7.53 ± 0.32b	4.44 ± 0.34bc	5.73 ± 0.2b	11.52 ± 0.47	5.07 ± 0.09	3.42 ± 1.18	1.73 ± 0.01	2.29 ± 0.05
92	1022	Toluene [J]	91	2		NF	Paint [a]	38.46 ± 1.4a	4.29 ± 0.86b	4.4 ± 0.25b	4.78 ± 0.32b	4.72 ± 0.18b	5.03 ± 0.1	1.71 ± 0.08	2.35 ± 0.16	3.52 ± 0.29	2.77 ± 0.09
93	1757	Naphthalene [W,J]	128	1		NF	Tar [a]	16.47 ± 0.65a	3.25 ± 0.12b	2.28 ± 0.1c	2.47 ± 0.03c	2.52 ± 0.03c	18.18 ± 0.7	3.17 ± 0.1	2.4 ± 0.12	2.86 ± 0.02	2.87 ± 0.03
94	1895	2-Methyl naphthalene [W,J]	142	2		NF	NF	15.68 ± 0.54a	3.24 ± 0.11b	2.47 ± 0.07c	NF	NF	18.14 ± 0.7	3.23 ± 0.11	2.54 ± 0.24	2.86 ± 0.03	2.85 ± 0.03
Pyrazines								-									
95	1385	2-Ethyl-6-methyl, pyrazine [W,J]	121	1	MR	NF	Nutty, grassy [b]	29.19 ± 0.97a	1.09 ± 0.09c	1.65 ± 0.04c	4.87 ± 0.43b	1.58 ± 0.4c	3.06 ± 0.08	0.68 ± 0.28	1.19 ± 0.08	0.39 ± 0.01	0.95 ± 0.03
96	1435	2,6-Diethyl, pyrazine [W,J]	135	1	MR	6[6]	Roasted, nutty [b]	NF	3.56 ± 0.47b	1.35 ± 0.09c	9.76 ± 1.14a	2.52 ± 0.39c	NF	NF	NF	NF	NF
97	1462	5-Ethyl-2,3-dimethylpyrazine [N]	135	1	MR	3[1]	Nutty roasted, woody [e]	NF	NF	1.57 ± 0.05bc	7.18 ± 3.13a	2.56 ± 0.31b	NF	NF	NF	NF	NF
98		2,3-diethyl,pyrazine [N]		1	MR	NF	NF	NF	NF	NF	NF	NF	NF	NF	1.23 ± 0.04	NF	1.35 ± 0.05
Phenols								-									
99	2010	Phenol [W,J]	95	1		5900[3]	Medicinal [a, d]	5.16 ± 0.17a	NF	1.06 ± 0.01c	1.29 ± 0.1b	0.85 ± 0.04d	2.25 ± 0.19	0.25 ± 0.01	0.63 ± 0.03	0.32 ± 0	0.64 ± 0.02
100	2146	4-ethenyl-2-methoxyphenol J	135	2		3[3]	Spicy, curry [a, f]	1.81 ± 0.38a	NF	NF	NF	NF	4.09 ± 0.09	1.14 ± 0.12	2.48 ± 0.22	1.05 ± 0.01	1.14 ± 0.12

OTV (odor threshold values; parts per billion, μg/L) in water were reported by: (1 Ho et al., [16]; 2 Jiang & Zhang, 2010; 3 Leffingwell & Associates, [28]; 4 Nan et al., 2013; 5 Qian & Wang, [39]; 6 Wang et al., [5]; 7 Wu et al., [19]; 8 Welke et al., 2014). Aroma descriptors were obtained from "Flavornet and human odor space" (a http://www.flavornet.org/flavornet.html), the LRI and odor data base (b http://www. odour.org.uk/odour/index.html) and from the reported literature (c Jiang & Zhang, 2010; d Qian & Wang, 2005; e Wang et al., 2017; f Welke et al., 2014; g Wu et al., 2016). "NF" indicated not found. Retention indices (RI): Kovats retention indices were calculated based on a n-alkane series (C6–C24) on the poly (ethylene glycol) (PEG) column under the same chromatographic conditions. G = grapes; R = raisin. Identification method (ID-M): 1, identified, mass spectrum and RI were in accordance with standards; 2, tentatively identified, mass spectrum matched in

the standard National Institute of Standards and Technology NIST 2008 library and RI matched with theNIST Standard Reference Database (NIST Chemistry WebBook). Ion (*m*/*z*): The characteristic ion (*m*/*z*) was used for choosing the corresponding compound and evaluating the peak areas of them in order to avoid. B: reported in Buttery, Seifert, Ling, Soderstrom, and Yerington [22] for the study of Thompson seedless raisins. W: reported in Wang et al. [2,3] for the study of sun- and air-dried raisins in different seedless varieties. J: reported in Javed et al. [13] for the study of raisin storage. NF: never reported as volatile compounds in Thompson seedless raisins.
